# Clinical risk factors for moderate and severe antituberculosis drug-induced liver injury

**DOI:** 10.3389/fphar.2024.1406454

**Published:** 2024-07-23

**Authors:** Quanxian Liu, Lu Huang, Hong Yan, Zhaojing Zong, Zhenyong Chen, Xiaoyan Wu, Ling Chen, Yuanbo Lan

**Affiliations:** Department of Tuberculosis, Affiliated Hospital of Zunyi Medical University, Zunyi, China

**Keywords:** drug-induced liver injury, moderate and severe ATB-DlLl, risk factor, malnutrition, haemoglobin

## Abstract

**Objective:**

To analyze the clinical and laboratory characteristics and to identify predictors of moderate to severe anti-tuberculosis drug-induced liver injury (ATB-DILI) in patients with tuberculosis.

**Methods:**

This prospective study enrolled Tuberculosis (TB) patients treated with first-line anti-tuberculosis drugs at the Affiliated Hospital of Zunyi Medical University between May 2022 and June 2023. The occurrence of ATB-DILI was monitored, and demographic and clinical data were gathered. We analyzed risk factors for the development of moderate to severe ATB-DILI.

**Results:**

ATB-DILI was detected in 120 (10.7%) of the patients, with moderate to severe ATB-DILI occurring in 23 (2.0%) of the 1,124 patients treated with anti-tuberculosis treatment. Multivariate cox regression analysis identified malnutrition (HR = 4.564, 95% CI: 1.029–20.251, *p* = 0.046) and hemoglobin levels <120 g/L (HR = 2.825, 95% CI: 1.268–11.540, *p* = 0.017) as independent risk factors for moderate to severe ATB-DILI.

**Conclusion:**

The incidence of moderate to severe ATB-DILI was found to be 2.0%. Malnutrition and hemoglobin levels below 120 g/L emerged as significant independent risk factors for the occurrence of moderate to severe ATB-DILI in this patient population.

## 1 Introduction

Tuberculosis (TB), an ancient infectious disease is caused by infection with the *Mycobacterium tuberculosis* (*MTB*) complex. It ranks as the world’s leading cause of infectious diseases and is among the 13 primary causes of death globally (WHO, 2023), posing a serious threat to public health. Annually, there are approximately 10.6 million new tuberculosis cases are reported worldwide (WHO, 2023), according to global estimates, the latest global TB report indicates that in 2022, roughly 1.3 million individuals are expected to die from TB, a figure nearly twice as high as the number of deaths attributed to HIV/AIDS (WHO, 2023), Furthermore, tuberculosis follows COVID-19 as the infectious disease with the second-highest mortality rate. The TB situation in China is particularly concerning, with the country ranking third worldwide in terms of TB burden. In 2022, China is estimated to have 748,000 new TB patients and 30,000 TB-related deaths. The national TB incidence and mortality rates are projected to be 52 and 2.0 cases per 100,000 individuals, respectively (WHO, 2023). Clearly, tuberculosis remians a serious threat to human health globally, and particularly within China.

Anti-tuberculosis chemotherapy is the primary treatment for tuberculosis (Dartois and Rubin, 2022). The first-line drugs, including isoniazid (H), rifampicin (R), pyrazinamide (Z) and ethambutol (E), are commonly used, and the WHO recommends the 2HRZE/4HR regimen as a treatment option for sensitive TB (Who, 2022). Unfortunately, some patients may experience anti-tuberculous drug-induced liver injury (ATB-DILI) during treatment (Devarbhavi et al., 2021), which can lead to hospitalization, discontinuation or restriction of the use of other effective antituberculous drugs in moderate to severe cases. This may result in therapeutic deficiencies (Medina-Caliz et al., 2016; Kullak-Ublick et al., 2017), changes in treatment regimen, prolongation treatment duration, and in hepatic failure (Gafar et al., 2019). Liver transplantation is the most effective treatment for liver failure, yet it is limited due to the scarcity and the cast of liver donors (Ling et al., 2022). Therefore, moderate to severe ATB-DILI not only poses a significant public health concerns but also imposes a substantial financial burden on society. Data indicated that among hospitalized patients with ATB-DILI, 20.1% experience severe liver injury, with a mortality rate of 34.5% due to liver failure during follow-up (Zhao et al., 2020); approximately 35.5% of hospitalized patients present with acute liver failure (ALF), and 9.7% of these patients will die (Wang et al., 2020). Even with prophylactic measures, a considerable number of patients still develop severe ATB-DILI and even die after drug administration (Naidoo et al., 2015; Mehta et al., 2021). This shows that the correct identification of factors for the development of moderate to severe ATB-DILI and identifying appropriate antituberculosis treatment regimens are important for the control of tuberculosis.

Previous research has identified risk factors for the development of moderate to severe ATB-DILI (Shen et al., 2014; Jiang et al., 2021; Zhong et al., 2021), but these studies often suffer from small sample sizes and limited consideration of exposure factors, leading to inconsistent or even contradictory conclusions. This has limited their clinical guidance. Therefore, our study prospectively followed a cohort of TB patients treated with the HRZE regimen to identify clinical risk factors for moderate and severe ATB-DILI. By early and precise identification of patients at high risk for moderate to severe ATB-DILI, we aim to assist clinicians in selecting the most appropriate antituberculosis treatment strategies.

## 2 Materials and methods

### 2.1 Study design

We enrolled tuberculosis patients who were treated with first-line anti-tuberculosis drugs at the Affiliated Hospital of Zunyi Medical University between May 2022 and June 2023. The primary objective was to observe the incidence of ATB-DILI and to analyze the risk factors associated with the development of moderate and severe ATB-DILI.

### 2.2 Inclusion and exclusion criteria

Inclusion criteria were as follows (had to be met simultaneously): 1) aged ≥18 years; 2) had active tuberculosis (including clinically diagnosed and laboratory-confirmed patients); 3) had an antituberculosis treatment regimen that included HRZ; and 4) had good adherence to medication and follow-up and voluntary participation in this study. Exclusion criteria were as follows: 1) had underlying liver disease, such as viral hepatitis B, cirrhosis, alcoholic hepatitis, or fatty liver, or those with obvious abnormalities in underlying liver function; 2) were using other drugs that may cause abnormalities in liver function, such as acetaminophen, herbal medicines, antitumour drugs or immunosuppressants; 3) had severe cardiac, pulmonary or renal insufficiency; 4) had combined autoimmune diseases, malignant tumors or HIV infection; and 5) were pregnant.

### 2.3 Clinical characteristics

Demographic data (e.g., sex, age), etiological findings for tuberculosis (e.g., acid-fast smear staining, *Mycobacterium tuberculosis* culture, *Mycobacterium tuberculosis* nucleic acid test), and initial biochemical parameters (before antituberculous therapy and when ATB-DILI) were meticulously recorded. ATB-DILI was diagnosed based on the criteria established by an international panel of experts (Aithal et al., 2011), which include meeting at least one of the following conditions: (i) alanine aminotransferase (ALT) levels ≥5 times the upper limit of normal (ULN); (ii) Alkaline phosphatase (ALP) ≥2 × ULN, particularly with accompanying elevations in concentrations of 5′- nucleotidase or γ-glutamyl transpeptidase in the absence of known bone pathology driving the rise in ALP level; (iii) More than or equal to threefold elevation in ALT concentration and simultaneous elevation of bilirubin concentration exceeding 2× ULN. The severity of ATB-DILI was categorized as mild, moderate, severe or fatal, in accordance with the International Consensus Criteria (Aithal et al., 2011). According to the simplified RUCAM scoring system, patients with a total score ≥6 points can be diagnosed with ATB-DILI (Ashby et al., 2021). The clinical phenotype of ATB-DILI patients was calculated according to the R value [R value = (serum ALT/ALT ULN)/(serum AKP/AKP ULN)]. Cases were classified as “hepatocellular” if the R value was ≥5, “cholestatic” if the R value was ≤2, and “mixed” if the R value was 2.0–5.0.

Basic demographic information, including sex, age, ethnicity, height, weight, alcohol consumption (both past and current) and smoking status (past and current), was collected through questionnaire prior to the initiation of antituberculosis treatment. All study participants underwent routine blood tests, liver function tests and kidney function tests. In adults, a body mass index (BMI) of less than 18.5 kg/m^2^ is regarded as an indication of malnutrition (Sinha et al., 2021). A serum albumin level of less than 35 g/L serves as a traditional marker of nutritional status (Keller, 2019). Thus, in this study, patients with a BMI of less than 18.5 kg/m^2^ or a serum albumin level of less than 35 g/L prior to anti-TB therapy were considered malnourished. Follow-up, including routine blood tests, biochemistry and sedimentation tests, were scheduled every 2 weeks for the first 2 months of anti-tuberculosis drugs use. Following 2 months of treatment, the frequency of follow-up visits and tests were reduced to once a month. Patients were asked to seek medical attention and review the relevant indexes in case of changes in their conditions. This schedule continued until the end of the antituberculosis course. The examination results were extracted from the patients’ electronic medical records. Doctors meticulously documented any liver damage that occurred during treatment and adjusted the antituberculosis treatment plan as necessary, as well as administered hepatoprotective and symptomatic treatments, depending on the severity of the liver damage.

### 2.4 Ethical approval and participation consent

The study design received ethical approval from the Ethics Committee of the Affiliated Hospital of Zunyi Medical University [Approval No.: KLL-2022-735]. Written informed consent was obtained from each subject to participate in this study. However, all the data were anonymous, and the confidentiality of the data was maintained. This study adhered to the Declaration of Helsinki.

### 2.5 Statistical data analysis

Statistical analyses were performed using SPSS 26.0 (SPSS Inc.) and GraphPad Prism 8.0.1 (GraphPad Software). For continuous variables, the mean (x) ± standard deviation (s) is used to represent the normal distribution. Otherwise, median and interquartile range were used. Categorical variables were expressed as percentages. Comparisons between groups were made using the parameterized Student’s t-test or the nonparametric Mann‒Whitney *U* test (continuous variables) and the χ2 test or Fisher’s exact test (categorical variables). Univariate and multivariate cox regression analyses was used to determine the independent risk factors for ATB-DILI and moderate and severe ATB-DILI. Variables with a *p*-value less than 0.2 in univariate cox regression analyses and factors considered significant by clinical experts were included in multivariate cox regression analyses by a backward-forward approach to identify risk factors for moderate and severe ATB-DILI. For multivariate cox analyses, the stepwise entry and removal probabilities were set to 0.05 and 0.10 respectively. Differences were considered statistically significant when *p* < 0.05.

## 3 Results

### 3.1 The clinical characteristics of ATB-DILI before ATB-DILI

**FIGURE 1:**
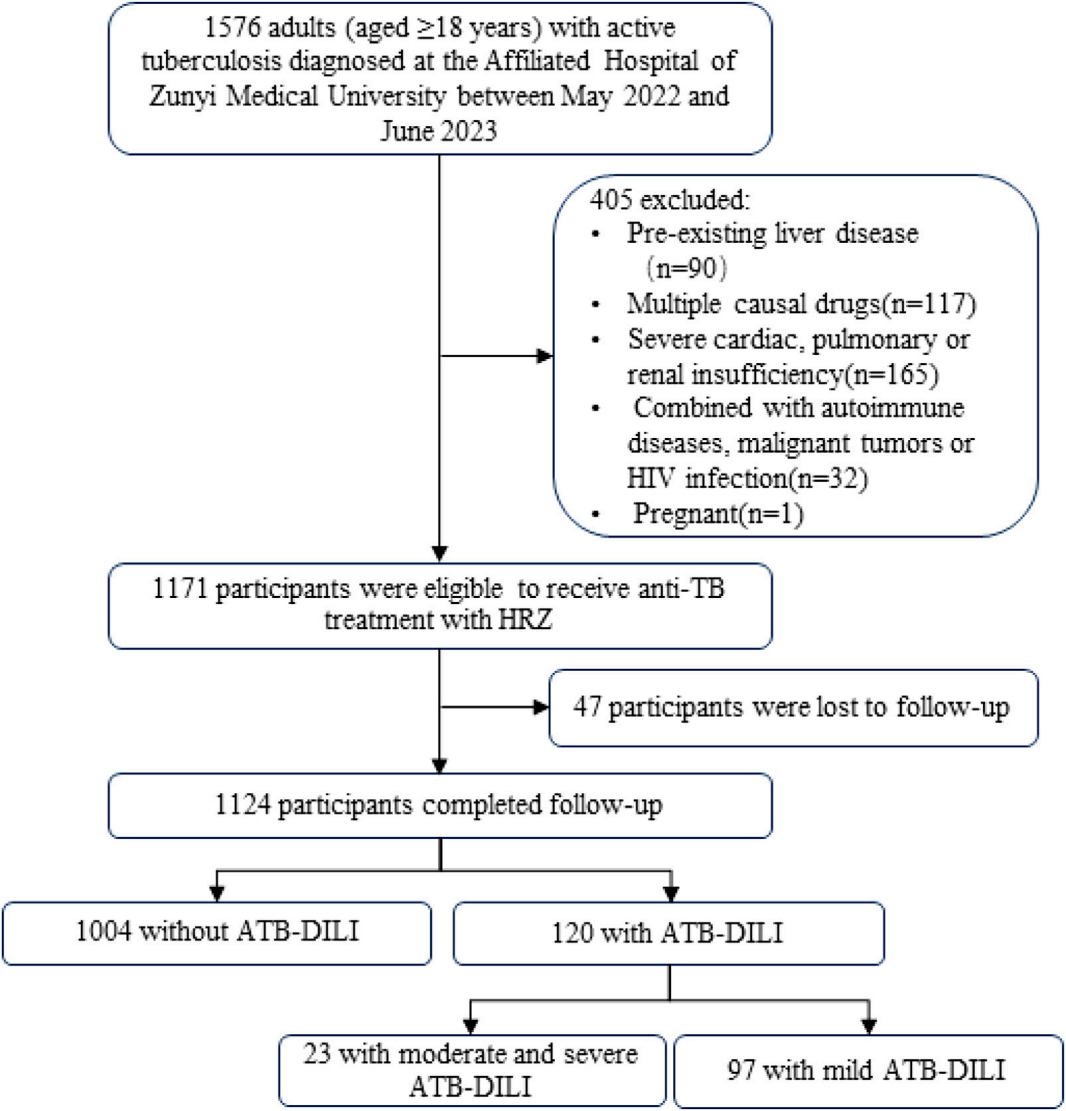
Participant flow chart HIV, human immunodeficiency virus; TB, tuberculosis; H, isoniazid; R, rifampicin; Z, pyrazinamide; ATB-DILI, anti-tuberculous drug-induced liver injury.

As shown in Figure 1, out of the 1,576 patients initially screened, a total of 1,124 patients who were treated with first-line anti-tuberculosis drugs were followed up until the end of the anti-tuberculosis treatment regimen. During this follow-up period, ATB-DILI was diagnosed in 120 patients. There were no statistically significant differences in mean age and BMI between patients with and without ATB-DILI. However, a lower percentage of patients with ATB-DILI were smokers compared to those without ATB-DILI (20.83% vs. 29.88%, *p* = 0.043). Before antituberculosis treatment, total lymphocyte count, ALT, aspartate transaminase (AST), gamma glutamyl transferase (GGT), and total bilirubin levels were significantly higher in patients with ATB-DILI than in those without the condition. Additionally, the hemoglobin concentration was significantly lower in patients with ATB-DILI compared to those without it [(123.56 ± 21.73) g/L vs. (127.78 ± 20.26) g/L, *p* = 0.043] (Table 1).

**TABLE 1:** Baseline characteristics of ATB-DILI patients versus non-ATB-DILI patients.

Characteristic	ATB-DILI (N = 120)	Non-ATB-DILI (N = 1,004)	*p*-value
Sex, n (%)			0.436
Male	73 (60.83)	572 (56.97)	
Female	47 (39.17)	432 (43.03)	
Ethnicity, n (%)			0.187
Han	113 (94.17)	904 (90.04)	
Non-Han	7 (5.83)	100 (9.96)	
Age (years)	45.48 ± 17.98	48.23 ± 18.21	0.117
Age group, n (%)			0.491
<65 years	96 (80.00)	770 (76.69)	
≥65 years	24 (20.00)	234 (23.31)	
BMI (kg/m2)	20.78 ± 2.63	20.82 ± 3.12	0.911
BMI group, n (%)			0.352
<18.5	77 (64.17)	688 (68.53)	
≥18.5	43 (35.83)	316 (31.47)	
Smoking, n (%)			**0.043**
No	95 (79.17)	704 (70.12)	
Yes	25 (20.83)	300 (29.88)	
Alcohol consumption, n (%)			0.096
No	88 (73.33)	803 (79.98)	
Yes	32 (26.67)	201 (20.02)	
Therapeutic category, n (%)			0.562
Initial treatment	111 (92.50)	940 (93.63)	
Retreatment	9 (7.50)	64 (6.37)	
Location of tuberculosis, n (%)			0.303
Intrapulmonary	113 (94.17)	914 (91.04)	
Extrapulmonary*	7 (5.83)	90 (8.96)	
Hypertension, n (%)			0.725
No	109 (90.83)	922 (91.83)	
Yes	11 (9.17)	82 (8.17)	
Diabetes, n (%)			0.671
No	115 (95.83)	949 (94.52)	
Yes	5 (4.17)	55 (5.48)	
Peripheral white blood cell count [×10^∧^9/L]	7.02 ± 2.97	6.91 ± 2.67	0.658
Number of neutrophil [×10^∧^9/L]	4.48 (3.24–5.32)	4.65 (3.26–5.44)	0.948
Monocyte count [×10^∧^9/L]	0.55 ± 0.25	0.56 ± 0.26	0.682
Total lymphocyte count [×10^∧^9/L]	1.30 (1.01–1.69)	1.28 (0.85–1.54)	**0.022**
Eosinophil count [×10^∧^9/L]	0.12 (0.04–0.17)	0.12 (0.06–0.15)	0.140
Platelet count [×10^∧^9/L]	259.37 ± 102.74	270.57 ± 102.41	0.258
Hemoglobin [g/L]	123.56 ± 21.73	127.78 ± 20.26	**0.043**
Erythrocytes [×10^∧^12/L]	4.41 ± 0.54	4.29 ± 0.67	0.053
ALT [U/L]	21.11 (14–31)	18.50 (12–22)	**0.002**
AST [U/L]	27.47 (20.05–32.75)	26 (19–28)	**0.031**
AKP [U/L]	103 (79–131.10)	107 (76–129.21)	0.251
GGT [U/L]	92.24 (43–114.34)	48 (22.08–92.24)	**0.029**
Serum albumin [g/L]	39.80 ± 5.81	38.31 ± 6.22	0.113
Total bilirubin [μmol/L]	12.70 (9.33–15.90)	10.90 (7.60–14.69)	**0.003**
INR	0.94 ± 0.09	0.97 ± 0.10	0.213
Creatinine [μmol/L]	67.70 ± 14.73	71.01 ± 34.76	0.060
Erythrocyte sedimentation rate [mm/h]	29.33 (21.25–49.14)	30.01 (20.45–47.44)	0.137

ATB-DILI, anti-tuberculosis drug-induced liver injury; BMI, body mass index; *, including extrapulmonary tuberculosis, tuberculosis combined with extrapulmonary tuberculosis; ALT, alanine aminotransferase; AST, aspartate transaminase; AKP, alkaline phosphatase; GGT, gamma glutamyl transferase; INR, international normalized ratio; significant results (*p* < 0.05) are highlighted in bold.

Out of the 120 patients with ATB-DILI, 23 cases manifesting as moderate and severe ATB-DILI. The mean age of these patients with moderate and severe ATB-DILI was 51.09 ± 17.20 years, with 6 patients (26.09%) being 65 years of age or older and an 6 patients (26.09%)were female. The average body mass index (BMI) of the patients with moderate and severe ATB-DILI was 19.86 ± 1.98 kg/m2, and 20 patients (86.96%) had a BMI <18.5 kg/m2. The proportion of moderate and severe ATB-DILI patients with BMI lower than 18.5 kg/m2 was significantly higher than that observed in patients with mild ATB-DILI (86.96% vs. 58.76%, *p* = 0.014). The patients predominantly considered of Han Chinese individuals who were receiving TB treatment for the first time. 6 (26.09%) and 6 (26.09%) patients with TB had a history of smoking and alcohol consumption, respectively. In addition, 3 (13.04%) TB patients had hypertension. The platelet count of patients with moderate and severe ATB-DILI was significantly lower than that of patients with mild ATB-DILI [(210.68 ± 82.11) ×10^9/L vs. 270.91 ± 104.10) ×10^9/L, *p* = 0.011] (Table 2).

**TABLE 2:** Baseline characteristics of patients with mild ATB-DILI versus patients with moderate and severe ATB-DILI.

Characteristic	Moderate ATB-DILI and severe ATB-DILI (N = 23)	Mild ATB-DILI (N = 97)	*p*-value
Sex, n (%)			0.234
Male	17 (73.91)	56 (57.73)	
Female	6 (26.09)	41 (42.27)	
Ethnicity, n (%)			>0.999999
Han	22 (95.65)	91 (93.81)	
Non-Han	1 (4.35)	6 (6.19)	
Age (years)	51.09 ± 17.20	44.14 ± 17.97	0.096
Age group, n (%)			0.399
<65 years	17 (73.91)	79 (81.44)	
≥65 years	6 (26.09)	18 (18.56)	
BMI (kg/m2)	19.86 ± 1.98	21.00 ± 2.73	0.061
BMI group, n (%)			**0.014**
<18.5	20 (86.96)	57 (58.76)	
≥18.5	3 (13.04)	40 (41.24)	
Smoking, n (%)			0.569
No	17 (73.91)	78 (80.41)	
Yes	6 (26.09)	19 (19.59)	
Alcohol consumption, n (%)			>0.999999
No	17 (73.91)	71 (73.20)	
Yes	6 (26.09)	26 (26.80)	
Therapeutic category, n (%)			0.067
Initial treatment	19 (82.61)	92 (94.85)	
Retreatment	4 (17.39)	5 (5.15)	
Location of tuberculosis, n (%)			>0.999999
Intrapulmonary	22 (95.65)	91 (93.81)	
Extrapulmonary*	1 (4.35)	6 (6.19)	
Hypertension, n (%)			0.439
No	20 (86.96)	89 (91.75)	
Yes	3 (13.04)	8 (8.25)	
Diabetes, n (%)			0.582
No	23 (100.00)	92 (94.85)	
Yes	0 (0.00)	5 (5.15)	
Peripheral white blood cell count [×10^∧^9/L]	6.36 ± 1.67	7.18 ± 3.19	0.232
Number of neutrophil [×10^∧^9/L]	4.49 (2.86–5.77)	4.47 (3.25–5.3)	0.828
Monocyte count [×10^∧^9/L]	0.50 ± 0.18	0.56 ± 0.26	0.300
Total lymphocyte count [×10^∧^9/L]	1.28 (0.88–1.57)	1.38 (1.06–1.7)	0.207
Eosinophil count [×10^∧^9/L]	0.12 (0.01–0.18)	0.11 (0.04–0.17)	0.947
Platelet count [×10^∧^9/L]	210.68 ± 82.11	270.91 ± 104.10	**0.011**
Hemoglobin [g/L]	126.43 ± 19.43	128.09 ± 20.54	0.726
Erythrocytes [×10^∧^12/L]	4.38 ± 0.43	4.42 ± 0.56	0.770
ALT [U/L]	21.11 (11–34)	21.11 (14.5–30)	0.928
AST [U/L]	27.47 (25–39)	27 (20–28)	0.092
AKP [U/L]	110 (81–131.1)	101 (77.5–131.1)	0.485
GGT [U/L]	92.24 (65–126.4)	92.24 (36.08–116.3)	0.317
Serum albumin [g/L]	37.55 ± 6.51	40.33 ± 5.54	0.039
Total bilirubin [μmol/L]	15.4 (10.4–20)	11.7 (9.1–15)	0.020
INR	0.88 ± 0.12	0.96 ± 0.07	0.110
Creatinine [μmol/L]	71.63 ± 21.05	66.76 ± 12.76	0.154
Erythrocyte sedimentation rate [mm/h]	30.17 (24–50.17)	31.04 (20.5–48.56)	0.437

ATB-DILI, anti-tuberculosis drug-induced liver injury; BMI, body mass index; *, including extrapulmonary tuberculosis, tuberculosis combined with extrapulmonary tuberculosis; ALT, alanine aminotransferase; AST, aspartate transaminase; AKP, alkaline phosphatase; GGT, gamma glutamyl transferase; INR, international normalized ratio; significant results (*p* < 0.05) are highlighted in bold.

### 3.2 Incidence of ATB-DILI and clinical characteristics of patients with moderate and severe ATB-DILI at the onset of ATB-DILI

Among the 1,124 patients who completed anti-tuberculosis treatment, 120 patients developed ATB-DILI, including 23 patients (19.17%) with moderate and severe ATB-DILI and 97 patients (80.83%) with mild ATB-DILI. The overall prevalence of ATB-DILI was 10.7% (120/1,124), with a incidence rate of moderate and severe ATB-DILI was 2.0% (23/1,124). During the follow-up period, there were no deaths due to ATB-DILI.

Patients with moderate and severe ATB-DILI were significantly more likely experience jaundice than those with mild ATB-DILI (43.48% vs. 0%, *p* < 0.0001). There was no statistical difference in the incidence of antituberculosis drug allergy or the need for hospitalization between the moderate to severe ATB-DILI group and the mild ATB-DILI group. The majority of ATB-DILI cases, regardless of severity, occurred within the first 2 months of anti-TB therapy. However, the incidence of moderate to severe ATB-DILI between days 46–60 was significantly higher than that of mild ATB-DILI (34.78% vs. 9.28%, *p* = 0.0044). Regardless of the severity of ATB-DILI, the liver injury type was mainly hepatocellular injury type. After the occurrence of ATB-DILI, patients with moderate and severe ATB-DILI has significantly higher mean serum aspartate transaminase (AST) and glutamyl transferase (GGT) concentrations compared to those with mild ATB-DILI, 453 (228–699) U/L vs. 212.5 (117.5–330.25) U/L, *p* = 0.005 and 141.00 (76.50–256.25) U/L vs. 92.50 (57.00–156.50) U/L, *p* = 0.033, respectively. In addition, patients with moderate to severe ATB-DILI had significantly higher mean serum total bilirubin concentrations compared to those with mild ATB-DILI [66.40 (45.70–107.70) μmol/L vs15.10 (9.75–19.83) μmol/L. P < 0.0001] (Table 3).

**TABLE 3:** Clinical characteristics of patients with moderate and severe ATB-DILI at the time of liver injury compared to patients with mild ATB-DILI.

Characteristics	Moderate and severe ATB-DILI (n = 23)	Mild ATB-DILI (n = 97)	*P*
Clinical presentation, n (%)			
Jaundice	10 (43.48)	0 (0.00)	**<0.0001**
Hospital admission	20 (86.96)	75 (77.32)	0.3998
Hypersensitivity	1 (4.35)	3 (3.09)	0.5782
Occurrence time of ATB-DILI (days), n (%)			
0–14	5 (21.74)	35 (36.08)	0.2260
15–30	5 (21.74)	44 (45.36)	0.0577
31–45	1 (4.35)	3 (3.09)	0.5782
46-60	8 (34.78)	9 (9.28)	**0.0044**
>60	4 (17.39)	6 (6.19)	0.0974
Liver injury pattern, n (%)			
Hepatocellular	15 (65.22)	66 (68.04)	0.8079
Mixed	7 (30.43)	17 (17.53)	0.2435
Cholestatic	1 (4.35)	14 (14.43)	0.2976
R value	9.26 (4.48–14.87)	7.62 (4.52–14.54)	0.431
Peripheral white blood cell count [×10^9^/L]	5.52 (4.56–9.06)	5.65 (4.41–7.00)	0.473
Number of neutrophil [×10^9^/L]	4.37 (2.70–5.87)	3.55 (2.47–4.78)	0.207
Monocyte count [×10^9^/L]	0.51 (0.42–0.81)	0.45 (0.34–0.67)	0.250
Total lymphocyte count [×10^9^/L]	0.97 (0.05–1.45)	1.13 (0.52–1.77)	0.125
Eosinophil count [×10^∧^9/L]	0.16 (0.06–0.75)	0.12 (0.04–0.54)	0.409
Platelet count [×10^9^/L]	190.10 (143–297.5)	234.50 (178.5–277.5)	0.271
Hemoglobin [g/L]	123.62 ± 24.12	130.76 ± 21.09	0.181
Erythrocytes [×10^∧^12/L]	4.47 (3.35–4.95)	4.56 (4.09–5.04)	0.230
ALT [U/L]	384 (223–683)	280 (213.00–427.50)	0.069
AST [U/L]	453 (228–699)	212.5 (117.5–330.25)	**0.005**
AKP [U/L]	178.14 (116.00–219.00)	178.14 (102.00–193.70)	0.504
GGT [U/L]	141.00 (76.50–256.25)	92.50 (57.00–156.50)	**0.033**
Serum albumin [g/L]	34.40 (28.50–38.60)	37.25 (33.43–43.35)	0.064
Total bilirubin [μmol/L]	66.40 (45.70–107.70)	15.10 (9.75–19.83)	**<0.0001**
INR	1.08 (1.04–1.26)	1.03 (0.95–1.15)	0.095
Creatinine [μmol/L]	65.00 (56.25–71.78)	62.13 (52.50–77.00)	0.882
Erythrocyte sedimentation rate [mm/h]	17.50 (2.25–53.50)	16.00 (4.00–44.00)	0.859

ATB-DILI, anti-tuberculosis drug-induced liver injury; R value = [serum ALT/ALT ULN]/[serum AKP/AKP ULN]; ALT, alanine aminotransferase; AST, aspartate transaminase; AKP, alkaline phosphatase; GGT, gamma glutamyl transferase; INR, international normalized ratio; significant results (*p* < 0.05) are highlighted in bold.

### 3.3 Predictors of ATB-DILI and moderate and severe ATB-DILI

We performed univariate and multifactorial cox regression analyses to identify potential risk factors for the development of moderate and severe ATB-DILI, using baseline data from prior to the initiation of anti-TB therapy. Univariate cox regression analysis revealed that age ≥65 years, malnutrition (including BMI <18.5 kg/m^2^ or serum albumin <35 g/L) and hemoglobin <120 g/L were risk factors for ATB-DILI (Table 3). Multivariate cox regression analysis revealed that malnutrition (HR = 2.047, 95% CI: 1.279–3.276, *p* = 0.003), alcohol consumption (HR = 1.710, 95% CI: 1.033–2.832, *p* = 0.037) and hemoglobin <120 g/L (HR = 2.289, 95% CI: 1.353–3.870, *p* = 0.002)were independent risk factors for ATB-DILI in patients with tuberculosis (Table 4).

**TABLE 4:** Independent predictors of ATB-DILI.

Factor	Univariate				Multivariate			
	HR	95% CI		P	HR	95% CI		P
		Lower	Upper			Lower	Upper	
Female	0.915	0.596	1.404	0.683				
Age ≥65 years	1.790	1.031	3.106	**0.038**	1.263	0.674	2.366	0.465
Malnutrition*	1.732	1.116	2.688	**0.014**	2.047	1.279	3.276	**0.003**
Non-Han	0.537	0.218	1.326	0.178	0.635	0.254	1.589	0.332
Alcohol consumption	1.585	0.982	2.559	0.059	1.710	1.033	2.832	**0.037**
Smoking	0.937	0.544	1.615	0.815				
Hypertension	1.744	0.802	3.790	0.161	1.491	0.657	3.383	0.340
Diabetes	1.464	0.203	10.545	0.705				
Extrapulmonary tuberculosis	0.589	0.257	1.352	0.212				
Retreatment	1.404	0.676	2.920	0.363				
Hemoglobin <120 g/L	1.921	1.218	3.029	**0.005**	2.289	1.353	3.870	**0.002**
White blood cell >10 × 10^∧^9/L	1.068	0.465	2.456	0.876				
Platelet count >300 × 10^∧^9/L	1.235	0.754	2.025	0.402				
Creatinine >100 μmol/L	0.941	0.230	3.849	0.933				
Total bilirubin>20 μmol/L	1.552	0.874	2.757	0.134	1.510	0.833	2.739	0.175
ALT >40U/L	1.018	0.590	1.756	0.950				
AST >40U/L	1.567	0.896	2.739	0.115	1.434	0.785	2.620	0.241
INR	2.211	0.715	1.224	0.756				

HR, hazard ratio; 95% CI, 95% confidence interval; *, BMI <18.5 kg/m^2^ or serum albumin <35 g/L; INR, international normalized ratio. Significant results (*p* < 0.05) are highlighted in bold.

As shown in Table 5, univariate and multivariate cox regression analyses were performed for risk factors associated with the development of moderate and severe ATB-DILI, also using data from before the start of anti-tuberculosis therapy. Univariate cox regression analysis showed that malnutrition and creatinine >100 μmol/L were risk factors for moderate and severe ATB-DILI. Multivariate cox regression analysis showed that malnutrition (HR = 4.564, 95% CI: 1.029–20.251, *p* = 0.046) and hemoglobin <120 g/L (HR = 2.825, 95% CI: 1.268–11.540, *p* = 0.017) were an independent risk factors for moderate and severe ATB-DILI on first-line antituberculosis therapy.

**TABLE 5:** Independent predictors of moderate to severe ATB-DILI.

Factor	Univariate				Multivariate			
	HR	95% CI		*P*	HR	95% CI		*P*
		Lower	Upper			Lower	Upper	
Female	0.398	0.129	1.223	0.108	0.470	0.136	1.631	0.234
Age ≥65 years	1.381	0.385	4.958	0.621				
Malnutrition*	4.806	1.088	21.227	**0.038**	4.564	1.029	20.251	**0.046**
Non-Han	0.518	0.067	3.981	0.527				
Alcohol consumption	1.557	0.573	4.232	0.386	2.017	0.411	9.882	0.387
Smoking	1.650	0.574	4.740	0.353				
Hypertension	0.522	0.068	3.986	0.531				
Extrapulmonary tuberculosis	1.940	0.252	14.918	0.524				
Retreatment	1.459	0.461	4.620	0.520				
Hemoglobin <120 g/L	2.469	0.855	7.130	0.095	2.825	1.268	11.540	**0.017**
White blood cell >10 × 10^∧^9/L	0.045	0.000	348.078	0.497				
Platelet count >300 × 10^∧^9/L	0.302	0.069	1.323	0.112	0.516	0.118	2.255	0.380
Creatinine >100 μmol/L	9.681	1.123	83.478	**0.039**	7.119	0.825	61.429	0.074
Total bilirubin>20 μmol/L	0.891	0.286	2.773	0.842				
ALT >40U/L	0.896	0.286	2.808	0.850				
AST >40U/L	2.476	0.791	7.748	0.119	2.136	0.612	7.463	0.234
INR	0.979	0.673	1.103	0.997				

HR, hazard ratio; 95% CI, 95% confidence interval; *, BMI <18.5 kg/m^2^ or serum albumin <35 g/L; INR, international normalized ratio. Significant results (*p* < 0.05) are highlighted in bold.

### 3.4 Follow-up outcome

There were no deaths in this study during the entire follow-up period until the end of anti-TB treatment. Of the patients with ATB-DILI, 89.2% (107/120) had their anti-tuberculosis therapy interruption due to ATB-DILI. The duration of anti-tuberculosis therapy interruption was 11 (5–25) days. The TB treatment success rate was 90.2% (906/1,004) for participants without ATB-DILI, 81.7% (98/120) in those with ATB-DILI, and 73.9% (17/23) in those with moderate and severe ATB-DILI. The TB treatment success rate of patients with ATB-DILI was significantly lower than that of patients without ATB-DILI (*p* = 0.007). However, there was no significant difference in TB treatment success rate between moderate and severe ATB-DILI patients and ATB-DILI patients (*p* = 0.397).

## 4 Discussion

In China, antituberculosis drugs are a major cause of drug-induced liver injury (Shen et al., 2019). However, the exact mechanisms behind ATB-DILI remain elusive. Among the first-line anti-tuberculosis drugs, isoniazid, rifampicin and pyrazinamide are the main agents associated with ATB-DILI, while reports of liver injury from ethambutol are rare (LiverTox, 2012). The hepatotoxicity of isoniazid and pyrazinamide may be due to their respective metabolites (Lim WS. et al., 2023). Pyrazinamide is metabolized into pyrazinic acid and 5-hydroxypyrazinic acid, which can cause liver injury (Hussain et al., 2021). Isoniazid *in vivo* metabolites acetylnicotinazid and hydrazide cause liver injury (Metushi et al., 2016). Additionlly, H can be directly oxidized to active metabolites that valently bind to liver macromolecules, leading to liver damage through immune mechanisms (Jenner and Ellard, 1989; Wang et al., 2016). Rifampicin is belived to enhance the hepatotoxicity of other anti-tuberculosis drugs, for example, by providing an acetyl group to H, thereby accelerating its metabolism to acetylhydrazine and causing liver damage (Kim et al., 2017). Rifampicin can also affect bile acid synthesis and transport, leading to cholestasis and promoting liver injury (Zhuang et al., 2022). Beyond these mechanisms, intestinal flora disturbance and intestinal barrier disruption are believed to be involved in the occurrence and development of ATB-DILI. These factors can expose the liver to increased drug or bacterial metabolites, triggering a persistent inflammatory response that promotes liver damage (Chopyk and Grakoui, 2020).

This study revealed that the incidence of ATB-DILI was 10.7%, with a moderate and severe ATB-DILI incidence rate of 2.0%. This incidence rate of ATB-DILI is higher than the 5.4% reported in a previous Chinese study involving 4,562 adult TB patients (Jiang et al., 2021) and the 7.6% in a recent multicenter prospective study in South Korea (Lim J. et al., 2023). However, both the overall ATB-DILI incidence and the incidence of moderate to severe ATB-DILI were lower than the rates reported by Portuguese scholars, which were 44.2% and 28.3% respectively (Cavaco et al., 2022). In Brazil, the incidence of severe ATB-DILI among TB patients with HIV infection reaches as high as 22.1% (Tomich et al., 2015). The discrepancies in incidence rates may be attributed to several factors: 1) variation in diagnostic criteria for ATB-DILI; 2) differences in study populations; 3) variation in prescription habits, and some medical staff may provide preventive liver protection drugs to reduce the incidence of ATB-DILI and severe ATB-DILI; 4) different genetic polymorphisms in different races; 5) different regional factors; and 6) unique metabolomes and the influence of the microbiome. Previous studies have shown that the plasma metabolome and urine microbiome of patients with ATB-DILI and severe ATB-DILI have their own characteristics before liver injury (Wang MG. et al., 2022; Wu et al., 2022). Therefore, it is suggested that the metabolome and microbiome have an impact on the occurrence and severity of ATB-DILI.

The patients in this study developed anti-tuberculosis ATB-DILI overwhelmingly within 2 months therapy, suggesting that the monitoring of liver function indexes should be strengthened during the intensive period of antituberculosis treatment (2 months before antituberculosis treatment). Furthermore, early initiation of liver function monitoring liver function at an early stage (2 weeks after antituberculosis treatment), is beneficial for predicting the occurrence of ATB-DILI (Patterson et al., 2021) and avoiding missed diagnoses of ATB-DILI and adverse outcomes (Hayashi et al., 2015). The proportion of moderate and severe ATB-DILI occurring in 46–60 days is significantly higher than that of mild ATB-DILI, which means that attention should be paid to the monitoring of biochemical indexes of liver function during anti-tuberculosis process to detect severe liver injury early. Predominantly, the type of liver injury observed corresponded to hepatocellular injury, aligning with previous studies (Shen et al., 2019).

It is now widely accepted that old age, alcoholism, hepatitis virus infection or a combination of other liver diseases, malnutrition or hypoproteinaemia and HIV infection are risk factors for ATB-DILI (Ali et al., 2020; Chalasani et al., 2021; Devarbhavi et al., 2021; Jiang et al., 2021). There are few reports in the literature on whether the severity of ATB-DILI severity is related to age. However, it has been found that persistent liver biochemical abnormalities are common after drug-related liver injury in older patients (Fontana et al., 2015). In a retrospective study by Zhao et al. (2020) older age was not found to be a risk factor for severe ATB-DILI (Zhao et al., 2020). Our study also revealed that age ≥65 years do not appear to be at increased risk factor for moderate to severe ATB-DILI, though further studies are needed to confirm this conclusion. Alcohol consumption is recognized as an independent risk factor for severe ATB-DILI, which is consistent with the findings of Ji, who developed a nomogram model to predict the risk of drug-induced liver injury in anti-tuberculosis treatment patients (Ji et al., 2023). The more alcohol consumed, the higher the risk of alcohol-related liver injury and even death (Surial et al., 2021). The effects of hepatitis virus infection, other acute and chronic liver diseases and HIV infection on severe ATB-DILI were not investigated in this study because patients with hepatitis virus infection, other acute and chronic liver diseases and HIV infection were excluded. Tuberculosis combined with malnutrition is very common (Feleke et al., 2019), which have been identified as an important risk factor for the occurrence of ATB-DILI (Lin et al., 2021). There is a significant linear relationship between malnutrition and ATB-DILI (Zhao et al., 2023). Our study extends this understanding by demonstrating that malnutrition is not only a risk factor for ATB-DILI but also for its moderate and severe forms. The relationship between anemia and anti-tuberculosis drug-induced liver injury and its severity is rarely reported in the literature. This study found that hemoglobin <120 g/L is a risk factor for ATB-DILI, and it is also a risk factor for moderate and severe ATB-DILI. The reasons may be as follows: 1) Anemia directly leads to a decrease in systemic oxygen delivery, resulting in liver cell ischemia and hypoxia. 2) Malnutrition leads to decreased liver synthesis of plasma proteins (such as hemoglobin, etc.) and erythropoietin deficiency (Cheung et al., 2012), so anemia patients are often combined with malnutrition, and malnutrition is currently recognized as a risk factor for ATB-DILI.

A large multicenter retrospective study revealed that AST, total bilirubin, platelets, prothrombin time, and gender were not found to be correlated with biochemical nonresolution in patients with drug-induced liver injury (Wang CY. et al., 2022). However, our prospective study did not identify any correlation between AST, total bilirubin, platelets, prothrombin time, and gender with ATB-DILI or moderate and severe ATB-DILI. Future multi-center prospective large sample studies on AST, total bilirubin, platelets, prothrombin time, gender and their correlation with ATB-DILI or moderate and severe ATB-DILI are warranted.

Our research demonstrated that TB patients with malnutrition, and with hemoglobin <120 g/L are more likely to develop moderate and severe ATB-DILI after anti-TB treatment. To some extent, the occurrence of moderate and severe ATB-DILI post-antituberculosis treatment can be prevented by paying attention to these populations. The design of personalized antituberculosis treatment plans for these people can enhance patients compliance and improve efficacy of antituberculosis treatment.

There are several limitations to our study. First, while the prospective follow-up study included 1171 TB patients receiving first-line anti-TB therapy, the sample size for patients with moderate to severe ATB-DILI patients was insufficient. Thus, the conclusion drawn from this study should be interpreted with caution and might not be extrapolated to the wider perspective of patients with tuberculosis worldwide. Second, the study did not investigate the polymorphisms in genes related to drug metabolism, transport, antioxidant response, and immune response, which are known to influence ATB-DILI susceptibility. Future studies should integrate genetic factors with the exploration of risk factors for moderate to severe ATB-DILI. Third, the study did not account for the impact of underlying liver diseases (such as viral hepatitis B, cirrhosis, combined autoimmune diseases, malignant tumors and HIV infection) and pregnancy status all have an impact on moderate and severe ATB-DILI. The exclusion of these populations limits the study’s ability to fully understand the risk factors for moderate to severe ATB-DILI in these groups, necessitating future research. Fourthly, alcohol consumption data were self-reported through questionnaires, which may not capture the full extent of alcohol intake and could introduce bias in the results. Lastly, the absence of routine blood lipid testing among participants precluded the inclusion of blood lipids in the analysis of ATB-DILI and its risk factors for moderate to severe cases.

## 5 Conclusion

Our study showed that malnutrition and hemoglobin <120 g/L as risk factors for the development of moderate to severe ATB-DILI. For patients who exhibit these characteristics before antituberculosis treatment, medical staff should be more cautious, and it is recommended that patients with low hepatotoxicity receive antituberculosis treatment to avoid moderate to severe ATB-DILI.

## Data Availability

The original contributions presented in the study are included in the article/Supplementary Material, further inquiries can be directed to the corresponding authors.
